# Development and validation of a nutrition assessment questionnaire based on the social and behavior change model for adolescents in Ethiopia

**DOI:** 10.3389/fpubh.2025.1474815

**Published:** 2025-04-08

**Authors:** Fantahun Ayenew Mekonnen, Gashaw Andargie Biks, Telake Azale, Netsanet Worku Mengistu

**Affiliations:** ^1^Department of Epidemiology and Biostatistics, Institute of Public Health, College of Medicine and Health Sciences, University of Gondar, Gondar, Ethiopia; ^2^Department of Health Systems and Policy, Institute of Public Health, College of Medicine and Health Sciences, University of Gondar, Gondar, Ethiopia; ^3^Department of Health Education and Behavioral Sciences, Institute of Public Health, College of Medicine and Health Sciences, University of Gondar, Gondar, Ethiopia; ^4^Department of Human Nutrition, Institute of Public Health, College of Medicine and Health Sciences, University of Gondar, Gondar, Ethiopia

**Keywords:** social and behavior change, dietary behavior, questionnaire, validity, reliability

## Abstract

**Background:**

A reliable assessment of behavior change requires the use of a validated tool based on an appropriate behavior change model. Research on tools for assessing nutrition behavior change is limited.

**Objective:**

This study aimed to develop and validate a questionnaire for assessing plant-protein food consumption behaviors based on Pender’s behavior change model, specifically for adolescent girls in Ethiopia.

**Methods:**

A collection of items was generated by examining relevant behavior change theories and manuals, dietary guidelines, and literature focused on pulses’ food function, processing, and preparation. The items were examined for content and face validity. Exploratory factor analysis was performed after verifying its assumptions, such as the factorability of the instrument using Bartlett’s test of sphericity and Kaiser-Meyer-Olkin (KMO) measure of sampling adequacy. Eigenvalue and scree plot were used to determine the number of factors. Factor loadings and communalities were employed for item retention. Cronbach’s alpha was calculated to assess the reliability at the scale and dimension levels.

**Results:**

Of the 53 items analyzed, 29 items and 6 factors were retained. The overall scale-level reliability was measured at 0.7210, while the factor-level reliabilities were as follows: 0.69 for factor 1 (i48, i49, i50, i52, i53, i31, and i32), 0.67 for factor 2 (i7, i8, i9, i10, i12, i13, and i14), 0.63 for factor 3 (i23, i24, i25, i26, fi27, and i28), 0.31 for factor 4 (i4, i5, i40), 0.59 for factor 5 (i35, i36, and i37), and 0.58 for factor 6 (i18, i19, and i20).

**Conclusion:**

The tool has an acceptable scale-level reliability. The factors are theoretically meaningful and align with the recommendations. The tool can serve as a foundation for developing tools in related fields. However, it requires further refinement before it can be used as a standard tool.

## Background

Protein-energy undernutrition increases the risk of morbidity and mortality and contributes to growth and developmental failures. The most alarming consequence of undernutrition is its intergenerational impact, particularly on women’s reproductive outcomes. Undernourished mothers are more likely to give birth to low-weight babies, who may grow up to be malnourished adolescents or adults who face increased health and socioeconomic risks. Furthermore, female offspring may inherit this malnutrition (1–6). Despite its disastrous consequences, undernutrition remains a highly prevalent public health issue in underdeveloped countries, such as Ethiopia (7, 8).

Numerous factors contribute to protein-energy undernutrition. However, insufficient dietary intake, especially of animal protein, is the most important and immediate cause in sub-Saharan Africa. This is mainly because animal protein is unaffordable for the populations living in this region. In Ethiopia, the majority of the population spends at least a week or a month without access to animal-protein foods—particularly meat (2, 9, 10). Fortunately, plant proteins, especially pulses, have been shown to be high-quality proteins essential for the growth and development of children and adolescents. Therefore, it is highly recommended to be used as a substitute for animal proteins in such situations (11–15). Pulses are the most common crops grown in Ethiopia, and they are relatively affordable for the majority of Ethiopians (11, 16). Therefore, we planned to examine the effect of a nutrition education intervention on increasing the consumption of pulse-based foods. The intervention was intended to be guided by a Social and Behavior Change (SBC) model. In this context, identifying a behavior change theory that is appropriate for a specific behavior is a crucial step (17, 18). Although several behavior change theories exist, we selected Pender’s Health Promotion Model (19) because it comprehensively considers the various factors affecting dietary behavior change, such as individual attitudes, experiences, and cultural and interpersonal influences (20–24). Although this model has been used in various nutrition education intervention studies, these studies either did not validate the dimensions and items at all or the validation process lacked clarity and was conducted in a different context (25–27). Therefore, we aimed to develop and validate a plant-protein food consumption behavior assessment questionnaire based on Pender’s behavior change model for adolescents in Ethiopia. Pender’s Health Promotion Model includes components such as prior related behavior, personal factors (biological, psychological, and sociocultural), perceived benefits, perceived barriers, self-efficacy beliefs, activity-related affect, interpersonal influence, situational influence, immediate competing demands and preferences, and commitment to a plan of action (19).

## Methods

### Item pool development

After confirming that there was no validated instrument for a behavior change model related to adolescent dietary behavior in general and pulse-based consumption in particular in Ethiopia, we collated items by reviewing different literature such as behavior change theories, dietary guidelines, and literature dedicated to pulse food function, processing, and preparation (11, 19, 28–31). Pender’s model of health promotion concepts and constructs was used to guide the generation and organization of our item pool. The constructs considered were perceived benefits, perceived barriers, self-efficacy beliefs, activity-related affect, interpersonal influence, situational influence, immediate competing demands and preferences, and commitment to a plan of action (19). The items were rated on a 5-point Likert scale as follows: 1 = strongly disagree, 2 = disagree, 3 = neutral, 4 = agree, and 5 = strongly agree for constructs such as perceived benefits, perceived barriers, activity-related affect, and situational influence; 1 = very unconfident, 2 = unconfident, 3 = neutral, 4 = confident, and 5 = very confident for the self-efficacy belief construct; 1 = never, 2 = rarely, 3 = sometimes, 4 = often, and 5 = always for the interpersonal influence, and immediate competing demands and preferences constructs; and 1 = none, 2 = little, 3 = somewhat, 4 = fairly, and 5 = very much for the commitment to a plan of action construct. Separate instructions were provided for groups of items with distinct properties. Items were revised several times for their wording, understandability, organization, and response formatting. The pool of items with the respective constructs was then reviewed by five experts. This group consisted of two experts in Public Health Nutrition, two specialists in Health Education and Behavioral Sciences, and an epidemiologist. The experts were given a list of items with a cover page attached. The cover page provided information describing the problem area of focus: “improving the consumption of pulse-based foods by nutrition education guided by a behavior change theory,” Pender’s Health Promotion Model (HPM), and its components. The target population consisted of adolescent girls aged 15–19 years who were attending grades 9–12. The item relevance rating scale was defined as follows: 4 = very relevant, 3 = relevant, 2 = slightly relevant, and 1 = not relevant (32), against which the experts were expected to rate each item. The cover page also had points that guided the qualitative examination of the items, such as the comprehensiveness or coverage of the item pool, the appropriateness of the response categories, and the understandability, clarity, and wording of the items. A Delphi technique was used for the expert review of the items. We distributed the questions/items to the experts, instructing them to include the details of their reviews along with their ratings in the spaces provided. We collected their reviews and redistributed the reviews to experts to allow each expert to comment on the comments of the others. We again collected the comments and looked for any disagreements. Whenever there were disagreements among the experts regarding certain items, we sought to facilitate further exchange of comments only among the experts who disagreed. If the disagreement continued, a third expert was sought to resolve the issue.

We revised the items for expert textual review, and the content validity index (CVI) was calculated for the relevance rating score. Item-level content validity indices (I-CVI) and scale-level content validity indices (S-CVI) were calculated based on scores given for each item by the experts and then were compared against the minimum cutoff (0.9) recommended for items to stage to the next level (32, 33). The tool was then translated into the Amharic language by two translators, one language expert, and a subject matter expert, and it was then administered to 10 adolescent girls aged 15–19 years, attending grades 9–12 outside of the study area. Feedback from the respondents was incorporated, and the tool was then assessed for its psychometric properties.

### Psychometric testing

Data were collected through a self-administered data collection method from 256 adolescent female participants aged 15–19 years, attending grades 9–12 of Dabat Secondary School, Dabat district, Northwest Ethiopia. The adolescents were selected using systematic random sampling.

**Figure 1:**
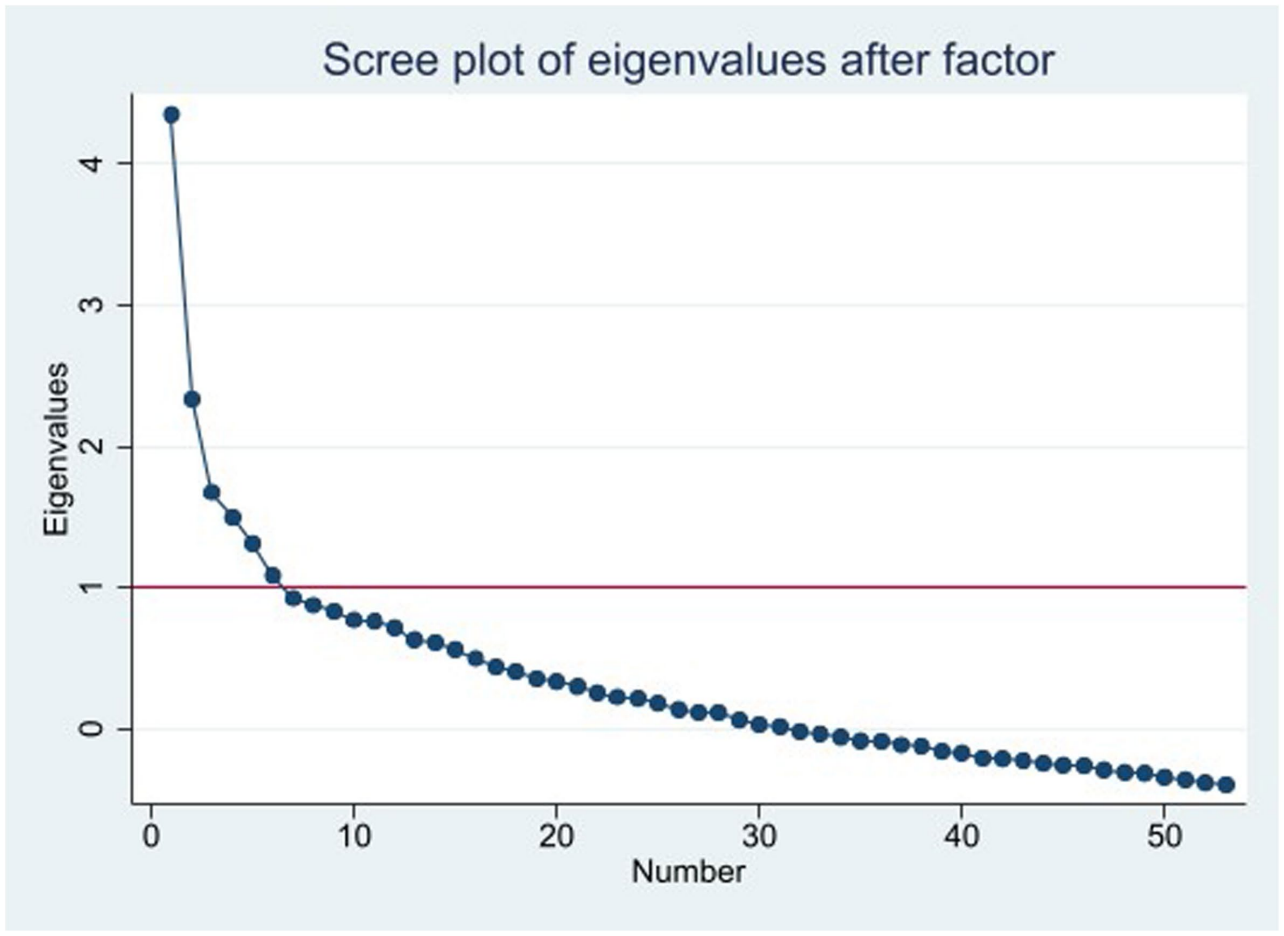
Factors retained for pulses-based food questionnaire for adolescents.

Psychometric analysis was conducted only on items that were found to be relevant according to expert ratings and target population interview feedback. A correlation matrix was produced using the Stata command correlate, as the items were on a 5-point Likert scale. Bivariate and multivariate normality and multicollinearity were checked using the histogram, the Shapiro–Wilk test, Doornik–Hansen and Mardia’s multivariate normality tests, and the Variance Inflation Factor (VIF), respectively (34). The appropriateness of the data for factor analysis was then assessed using Bartlett’s test of sphericity and the Kaiser-Meyer-Olkin (KMO) (44) measure of sampling adequacy. Bartlett’s test <0.05 and KMO > 0.6 were considered suggestive of factor analysis (35, 36). Eigenvalue and scree plot were used to determine the number of factors retained (36–38). A factor must load on at least three items, provided that there are no cross-loadings (34, 39–41). With regard to the retention of items, items with factor loading values >0.3 were retained. However, an item should not cross-load, and its commonality should not be <0.2 (34, 39). The factor loading was first used, and the commonality criterion was then followed to decide whether to retain the items or not. Finally, the scale-level and factor-level reliabilities of the items were determined by calculating Cronbach’s alpha. A Cronbach’s alpha above 0.7 was considered to indicate acceptable reliability (42, 43) (Figure 1).

## Results

### Sociodemographic characteristics of participants

A total of 256 adolescent girls aged 15–19 years attending grades 9–12 participated in this study. The participants aged 18 years accounted for the largest proportion of respondents, 77 (30.08%), while those aged 15 years accounted for the smallest proportion of respondents, 29 (11.33%). The total number of grade-9 participants was 68 (26.56%) and grade-12 participants were 56 (21.88%). The majority of the study participants, 139 (54.30%), were from rural areas. Almost all participants, 247 (96.48%), belonged to the Orthodox Christian religion (Table 1).

**Table 1:** Sociodemographic characteristics of adolescent girls in Northwest Ethiopia in October 2021 (*n* = 256).

Characteristics	Frequency (%)
Age (years)	
15	29 (11.33)
16	45 (17.58)
17	61 (23.83)
18	77 (30.08)
19	44 (17.19)
Grade	
9	68 (26.56)
10	71 (27.73)
11	61 (23.83)
12	56 (21.88)
Residence	
Urban	117 (45.70)
Rural	139 (54.30)
Religion	
Orthodox Christian	247 (96.48)
Protestant	1 (0.39)
Muslim	8 (3.13)

### Item pool development

For this study, a total of 63 items were generated. Of these, 10 items were removed based on expert suggestions that they were redundant, leaving a total of 53 items. Amendments were made to the remaining items. For instance, we changed the 3-point Likert scale to a 5-point Likert scale and merged the positive and negative feelings dimensions into activity-related affect dimensions. In terms of item and scale validity, the average item-level content validity index (I-CVI) was 94.6%, and the average scale-level content validity index (S-CVI) was 94.1%. The items were then translated from the English language into the respondents’ language, Amharic, by a language expert and an epidemiologist. The two bilingual translators—the methodology expert and the language expert—were not only experts but also had knowledge and experience of the culture of the respondents of the tool. Both experts brought their translations and discussed them item by item for any deviations. Fortunately, both experts agreed on all the translations. The items were then administered to 10 adolescent girls, and the feedback showed that the items were understandable and had no vague words or complex sentences.

### Psychometric testing

The examination of the assumptions related to the exploratory factor analysis of the data indicated no bivariate or multivariate normality. It also showed that there was no multicollinearity. Therefore, we used principal axis factoring as a method of factor extraction. The sampling adequacy and factor structure tests of the correlation revealed that there was an adequate correlation with Bartlett’s test of sphericity (*p* < 0.001) and Kaiser-Meyer-Olkin (KMO) (44) (0.64), suggesting factor analysis. The factor analysis with oblique rotation (promax) followed by sorting identified six factors according to Kaiser-Guttman criteria (Royal Statistical Society, London, UK) (eigenvalue >1) and scree plot elbow. The analysis resulted in a total of 39 items based on a factor loading cutoff of 0.3 or greater. However, two of these items (i22 and i29) had cross-loadings, and they were removed, leaving a total of 37 items. When examining for commonality, a total of eight items (i11, i16, i17, i21, i33, i34, i41, and i42) with commonality below 0.2 were removed, leaving a total of 29 items. The reliability of these 29 items was 0.7210. The factor-level reliability with the respective items was 0.69, 0.67, 0.63, 0.31, 0.59, and 0.58 for factor 1 (i48, i49, i50, i52, i53, i31, and i32), factor 2 (i7, i8, i9, i10, i12, i13, and i14), factor 3 (i23, i24, i25, i26, i27, and i28), factor 4 (i4, i5, and i40), factor 5 (i35, i36, and i37), and factor 6 (i18, i19, and i20), respectively (Table 2). The tool has good convergent but poor discriminant validities.

**Table 2:** Retained items and factors of pulse-based food consumption behavior assessment questionnaire for adolescents in Ethiopia (*n* = 256).

Items	Factor loading	Communality	Cronbach’s alpha (α)
Factor 1			0.6675
i48. I am committed to preparing and eating pulse-based foods in stew form	0.6723	0.4054	
i49. I am committed to preparing and eating pulse-based foods in roasted form	0.5776	0.3535	
i50. I am committed to preparing and eating pulse-based foods in boiled or sprouted form	0.3119	0.2198	
i52. I am committed to adding condiments to pulse-based foods to enhance their taste	0.5067	0.3663	
i53. I am committed to preparing and eating pulse-based foods mixed with rice	0.3675	0.2395	
i31. I like eating pulse-based foods in stew form	0.4214	0.37	
I32. I like eating pulse-based foods in roasted form	0.4866	0.39	
Factor 2			0.6657
i7. I do not eat pulse-based foods in boiled form since I do not like their taste	0.5169	0.2875	
i8. I do not eat pulse-based foods in sprouted form since I do not like their taste	0.4863	0.2505	
i9. I do not eat pulse-based food in roasted form since I do not like their taste	0.5253	0.3285	
i10. I do not eat pulse-based foods in bread/kita form since I do not like their taste	0.5780	0.3604	
i12. I do not eat pulse-based foods in boiled or sprouted form because they take a long time to prepare	0.3695	0.2119	
i13. Our family rarely prepares pulse foods in boiled or sprouted form	0.4071	0.2443	
i14. Our family rarely prepares pulse-based foods in bread/kita form	0.4216	0.2311	
Factor 3			0.6324
i23. I can plan to prepare and eat pulses in roasted form	0.3885	0.2053	
i24. I can plan to prepare and eat pulses in boiled form	0.5033	0.3084	
i25. I can plan to prepare and eat pulses in sprouted form	0.5100	0.3640	
i26. I can plan to prepare and eat pulses in bread/kita form	0.4353	0.2960	
i27. I can plan to prepare and eat pulses mixed with vegetables	0.4458	0.2800	
i28. I can plan to prepare and eat pulses mixed with rice	0.5257	0.3091	
Factor 4			0.4173
i4. Eating pulse-based foods can help prevent diseases	0.4558	0.2053	
i5. Eating pulse-based foods can help fasten growth and development	0.3589	0.196	
i40. Three is at least one family member eating pulse-based foods in boiled form	−0.3620	0.21	
Factor 5			0.5911
i35. My friends expect me to eat pulse-based foods daily	0.5297	0.2769	
i36. My family members encourage me to eat pulse-based foods daily	0.5696	0.3232	
i37. My friends encourage me to eat pulse-based foods daily	0.5366	0.3051	
Factor 6			0.5768
i18. Since vegetables are not accessible/expensive, I cannot eat pulses mixed with vegetables	0.5500	0.3315	
i19. Since I do not have knowledge/skills, I cannot prepare and eat pulses mixed with vegetables	0.5271	0.2777	
i20. Since I do not have knowledge/skills I cannot prepare and eat pulses mixed with rice.	0.4329	0.2236	
Overall			0.7210

Regarding the naming of the factors, factor 1 comprises items related to one’s commitments to prepare different types of pulse-based foods (e.g., I am committed to preparing and consuming pulse-based foods in boiled or sprouted form), so it received the name commitment to a plan of action; factor 2 and factor 6 are about the barriers to preparing and consuming different types of pulse-based foods. Factor 2 items are about pulse-based foods’ test-related barriers (e.g., I do not eat pulse foods in boiled form since I do not like their taste), while factor 6 is about knowledge/skills and accessibility-related barriers to preparing and consuming pulse-based foods mixed with vegetables and/or rice (Since I do not have the knowledge/skills, I cannot prepare and eat pulses mixed with rice). Factor 3 is about self-efficacy beliefs to prepare and consume different pulse-based foods (e.g., I can plan to prepare and eat pulses in roasted form). Factor 4 is about the perceived benefits of pulse-based foods (e.g., Eating pulse-based foods can help fasten growth and development), and factor 5 is about the interpersonal influence of eating pulse-based foods (e.g., My friends expect me to eat pulse-based foods daily).

## Discussion

The aim of this study was to develop and validate a plant-protein food consumption behavior assessment questionnaire based on Pender’s behavior change model for adolescents in Ethiopia. The validation study retained a total of 29 items with acceptable reliability. It has also identified six factors and five components. The six factors included factor 1 (commitment to prepare and consume different types of pulse-based foods), factor 2 (barriers to preparing and eating different types of pulse-based foods—pulse-based foods’ taste-related barrier—specifically), factor 3 (self-efficacy beliefs to preparing and eating different pulse-based foods), factor 4 (perceived benefit of pulse-based foods), factor 5 (interpersonal influence for eating pulse-based foods), and factor 6 (barriers to preparing and eating different types of pulses-based foods, knowledge/skills, and accessibility-related barriers to prepare and consume pulse-based foods, specifically). Factor 2 and factor 6 are about barriers to preparing and eating pulse-based foods. However, although they both are barriers, the items in each factor belong to a similar category of barriers. One category of items (factor 2) was about pulse-based foods’ taste-related barriers to preparing and consuming pulse-based foods, while the other category of items (factor 6) was about knowledge/skills and accessibility-related barriers to preparing and consuming pulse-based foods. It seems logical to have subcategories of components whenever a component has distinct aspects. Factor 2 is related to pulse-based foods and factor 6 is related to individual person’s attributes. Literature shows that unlike cereals and animal-based foods, the palatability of pulse-based foods is not appealing, so they are usually eaten with condiments or spices (11).

The current study retained five out of the eight components of Pender’s HPM, which included perceived benefits, perceived barriers, self-efficacy beliefs, activity-related affect, interpersonal influence, situational influence, immediate competing demands and preferences, and commitment to a plan of action (19) even though all 63 items were generated and organized under each Pender’s HPM components. The components identified in the current study were perceived benefits, perceived barriers (factors 2 and 6), self-efficacy beliefs, interpersonal influence, and commitment to a plan of action. The components that were eliminated, in contrast, were activity-related affect, situational influence, and immediate competing demands and preferences. They were eliminated because they had no item that fulfilled either the factor loading or the commonality cutoff values. All the items generated for and organized under these components were eliminated for not fulfilling the criteria.

On the contrary, three items were found to form clusters with items of different concepts.

This tool validation study is the first of its kind to validate nutrition-related tools based on a behavior change model. The items were comprehensive, spanning the different contributors of dietary behavior in adolescents. However, the scale and factor-level reliabilities may have been compromised due to the small sample size.

## Conclusions and recommendations

This study identified a 29-item scale with an acceptable level of reliability and six factors. The factors identified are theoretically relevant and consistent with the recommendations. Therefore, the tool can be used as a springboard to develop and validate new tools in related fields. However, we also recommend that future studies should consider confirmatory factor analysis.

## Data Availability

The raw data supporting the conclusions of this article will be made available by the authors, without undue reservation.
